# Differential Expression Profiling Reveals Stress-Induced Cell Fate Divergence in Soybean Microspores

**DOI:** 10.3390/plants9111510

**Published:** 2020-11-07

**Authors:** Brett Hale, Callie Phipps, Naina Rao, Asela Wijeratne, Gregory C. Phillips

**Affiliations:** 1 College of Science and Mathematics, Arkansas State University, Jonesboro, AR 72467-1080, USA; awijeratne@astate.edu; 2 Arkansas Biosciences Institute, Arkansas State University, Jonesboro, AR 72467-0639, USA; callie.phipps@smail.astate.edu (C.P.); naina.rao@outlook.com (N.R.); gphillips@astate.edu (G.C.P.); 3 College of Agriculture, Arkansas State University, Jonesboro, AR 72467-1080, USA; 4 Agricultural Experiment Station, University of Arkansas System Division of Agriculture, Jonesboro, AR 72467-2340, USA

**Keywords:** microspore embryogenesis, totipotency, microgametogenesis, cell fate, RNA-Seq, soybean

## Abstract

Stress-induced microspore embryogenesis is a widely employed method to achieve homozygosity in plant breeding programs. However, the molecular mechanisms that govern gametophyte de- and redifferentiation are understood poorly. In this study, RNA-Seq was used to evaluate global changes across the microspore transcriptome of soybean (*Glycine max* [L.] Merrill) as a consequence of pretreatment low-temperature stress. Expression analysis revealed more than 20,000 differentially expressed genes between treated and control microspore populations. Functional enrichment illustrated that many of these genes (e.g., those encoding heat shock proteins and cytochrome P450s) were upregulated to maintain cellular homeostasis through the mitigation of oxidative damage. Moreover, transcripts corresponding to saccharide metabolism, vacuolar transport, and other pollen-related developmental processes were drastically downregulated among treated microspores. Temperature stress also triggered cell wall modification and cell proliferation—characteristics that implied putative commitment to an embryonic pathway. These findings collectively demonstrate that pretreatment cold stress induces soybean microspore reprogramming through suppression of the gametophytic program while concomitantly driving sporophytic development.

## 1. Introduction

Nonzygotic embryogenesis provides an excellent platform for the study of cell fate determination in plants [1]. This is well-illustrated by androgenesis, a phenomenon reliant upon the acquisition of totipotency and embryogenic competence within the male gametophyte (i.e., the microspore). In vitro, androgenesis is initiated oftentimes by subjecting microspores/bicellular pollen to exogenous stress, prompting callogenesis/direct embryogenesis if succeeded by an appropriate tissue culture regimen [2]. The resultant embryos and plants pose a source of diverse, genetically fixed material representative of meiotic recombination in the founder cell. Thus, these tissues are exploited for cultivar development in responsive species, and their remarkable display of cellular plasticity is used to study stress-induced histodifferentiation and cell cycle regulation [3,4]. To date, androgenesis protocols have been reported for more than 250 plant species [5].

The microspore is terminally differentiated in vivo, being destined to undergo microgametogenesis and to give rise to progeny in a fertilization-dependent manner. Diversion from this developmental pathway, whether spontaneous or induced, is accompanied by cytological and molecular attributes that contrast those of microspores with a gametophytic cell fate. A prominent phenotypic marker of cellular dedifferentiation is altered symmetry during the first mitotic division that inhibits daughter cell specialization and may ultimately render nonfunctional pollen [6]. Synchronously, totipotent/embryogenic microspores are denoted by cytoskeletal rearrangement, abnormal nuclear migration, fragmentation of the vacuole, and other characteristics that collectively present a “star-like” morphology [7,8,9]. Early embryogenic response is followed by rapid cell division and dehiscence of the microspore exine [10]. The resulting pro-embryo then undergoes callogenesis, zygotic-like embryogenesis, or developmental arrest, with determinants for embryo vs. callus cell fate remaining largely unknown. One hypothesis for microspore-derived callogenesis is an inadequate culture regimen, which when corrected, promotes direct embryogenesis [2]. This concept is supported by a historical increase in the embryo-to-callus ratio in model species (e.g., *Brassica napus* L. [2]); however, it does not provide a straightforward solution to achieve direct embryogenesis, nor does it credit the utility of callogenesis (and subsequent organogenesis) for regeneration in recalcitrant systems (e.g., the Cucurbitaceae [11]). Recent studies have emphasized the vital role of intrinsic molecules for microspore embryogenesis, including structural compounds (i.e., arabinogalactan proteins [12]), phytohormones [13], and calcium [14]. A heightened understanding of such components, as well as their modulation through tissue culture, will likely prove critical for increasing the morphogenic response in nonmodel androgenesis systems.

Redirection of microspore cell fate is characterized by three overlapping molecular programs: (i) mitigation of cellular damage in the presence of stress factors; (ii) induction of totipotency through the deregulation of gametogenesis; and (iii) specification of embryo identity [2,15]. Each developmental state is driven by transcriptional, post-transcriptional (e.g., small RNA (sRNA) patterning [1,16]), and post-translational (e.g., DNA methylation [17]; histone methylation/acetylation [18,19]) modifications that play a role in the transition from gametophytic to sporophytic growth. Gene expression studies have shed light on key aspects of this developmental switch, yet a holistic understanding of microspore reprogramming, particularly in its earliest stages, remains to be elucidated. Existent knowledge gaps are attributed to many factors, including (i) culture heterogeneity (in terms of both microspore developmental stage and response); (ii) concurrent expression of pollen and embryo transcripts within the same cell; (iii) variable inductive treatment between species; and (iv) recalcitrance in the model plant *Arabidopsis thaliana* L. [1,12,20,21]. Furthermore, transcriptome-wide analysis of microspores post-induction has only been employed within the Poaceae [1,22] and Brassicaceae [23]. Additional studies are needed to fully understand the molecular basis of totipotency in less-responsive species, as well as to conclusively link genes to cell fate determination rather than stress response.

Cultivated soybean (*Glycine max* [L.] Merrill) is a fabaceous crop that demonstrates recalcitrance to androgenic stimuli [24]. Recently, Garda et al. [25] reported a soybean tissue culture regimen that yielded sporophytic structures of microspore origin. This entailed a donor plant pretreatment, a nitrogen starvation basal medium (constructed from the works of Nitsch and Nitsch [26], Gamborg [27], and Lichter [28]), and the use of exogenous phytohormones at levels comparable to legume somatic embryogenesis systems [29,30]. The study, in addition to complementary cytological analyses [31], suggested that pre-isolation low-temperature stress was essential to induce totipotency within the soybean microspore [25]. These findings coincide with the majority of androgenesis protocols in which temperature-triggered physiological changes are required for microspore embryogenesis to occur ([7,32] and references therein). Thus, the objective of the present study was to employ RNA-Seq to evaluate the soybean microspore transcriptome following pretreatment low-temperature stress. Expression analysis revealed 20,027 differentially expressed genes (DEGs) between the treated and control microspore populations, many of which corresponded to the three aforementioned molecular programs. Sequencing data were supported by microscopic analysis of stressed microspores cultured in vitro, some of which displayed organellar reorientation, starch reduction, and exine dehiscence before differentiating into tissues with meristem identity. The results herein support the utility of temperature stress for cell fate divergence in soybean microspores and further characterize early events during the induction of totipotency/embryogenesis in a recalcitrant plant species.

## 2. Results

### 2.1. Temperature-Stressed Microspores Presented a Nongametophytic Morphology

**Figure 1:**
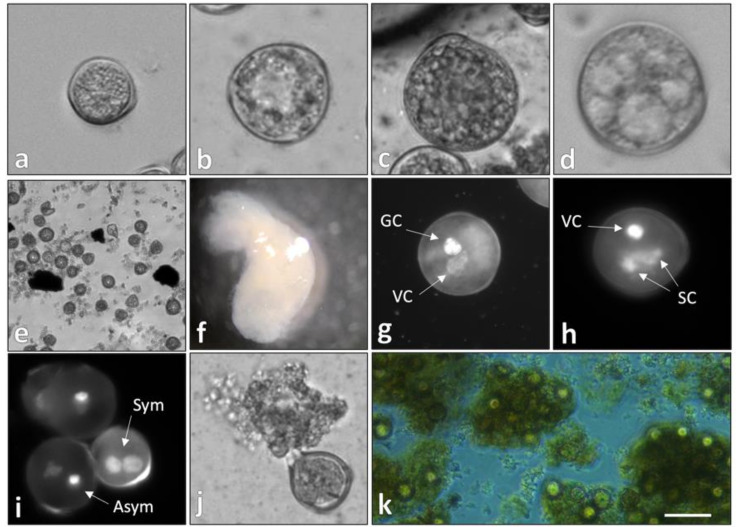
In vitro development of temperature-stressed soybean microspores. (**a**–**c**) Microgametogenesis, denoted by cell enlargement and the presence of starch granules; (**d**–**f**) microspore embryogenesis in soybean. (**d**) Embryogenic microspore with evident cytoskeletal fibers fragmenting the cytoplasm; (**e**) rapidly dividing pro-embryo; (**f**) microspore-derived embryo with established polarity; (**g**,**h**) pollen mitosis 1 (**g**) and 2 (**h**) observed in noninduced microspores; (**i**–**k**) cytological markers associated with an embryogenic culture. (**i**) Symmetrical division during pollen mitosis 1 observed via DAPI staining; (**j**,**k**) secretion of intrinsic molecules from nonisodiametric cells into the induction medium, forming a matrix. VC = vegetative cell; GC = generative cell; SC = sperm cell; Sym = symmetrical mitotic division; Asym = asymmetrical mitotic division. (**a**–**c**) bars = 15 μm; (**d**) 5 μm; (**e**) 100 μm; (**f**) 1 mm; (**g**,**h**) 10 μm; (**i**,**j**) 15 μm; (**k**) 100 μm.

The development of stressed, in vitro-cultured soybean microspores was evaluated through light and fluorescent microscopy in this study. Predominant phenotypes corresponded to cell death and microgametogenesis (Figure 1a–c) with the exception of a population diverted to a sporophytic pathway (Figure 1d–f). Characteristics observed exclusively within induced/sporophytic microspores included: (i) Reduction/absence of starch granules (Figure 1d); (ii) the presence of connective strands between the perinuclear and subcortical cytoplasm/vacuolar fragmentation (Figure 1d); (iii) rapid cell division/histodifferentiation (Figure 1e,f); and (iv) symmetrical division during the first pollen mitosis (Figure 1i). Stressed cultures also displayed a population of reduced, nonisodiamteric microspores that extruded endogenous molecules into the induction medium (Figure 1j,k), forming an opaque matrix that supported embryogenesis. The cytological markers observed here are consistent with those documented in model systems (reviewed in [7,21]) and have been described to a varying degree in other works of soybean androgenesis [25,31,33,34,35,36]. Such phenotypes were present largely after several days of culture; thus, microscopy alone was not sufficient to determine whether low frequencies of observation (<5% structures in Figure 1e; <1% structures in Figure 1f) were implicit of premature developmental arrest or a lack of induction.

While pre- and post-isolation conditions likely had a combinatorial effect on morphotype, some sporophytic characteristics (i.e., symmetrical mitotic division) were observed prior to culture, suggesting their induction via temperature stress. This contradicts the work of Moraes et al. [34] that reported no effect of pretreatment condition on the microspore fate divergence in soybean. Differences in observation may be due to the use of plants grown in a controlled environment in the present study vs. the use of field-grown materials by Moraes et al. [34], the latter of which was sub-optimal due to limited synchronization between microspore developmental stage and floral bud length [24,25]. In addition, the current study utilized the in-planta, 4 d stepwise pre-isolation temperature stress of Garda et al. [25] followed by a 1 wk post-culture acclimatization period (see Section 3.2), which greatly contrasted the induction regimen of the study in comparison (bud-only stress at 4 °C for 12 h [34]). It is, therefore, not unexpected to discern differences in androgenic response between the two works.

### 2.2. Cold Treatment Elicited a Classic Stress Response

Exogenous stress oftentimes triggers a cytoprotective molecular program that mitigates the accumulation of reactive oxygen species within the microspore. This entails the upregulation of genes encoding glutathione S-transferases, heat shock proteins (HSPs), and cytochrome P450s, among others [22,37,38,39]. In this study, RNA-Seq revealed the differential expression of 28 genes encoding members of the HSP70 family, 26 of which were upregulated in the stressed microspore population (Supplementary File 1). This was consistent with findings in *Hordeum vulgare* L. [22], *Brassica napus* L. [40], and Capsicum [41] in which HSP70s were spatiotemporally expressed following temperature pretreatment. In addition, stressed microspores demonstrated increased expression of HSP20-encoding genes (i.e., *Glyma.08g068700*), which was similar to observations within the microspore embryogenesis platforms of Bélanger et al. [22] and Zarsky et al. [42]. The role of HSP synthesis beyond cytoprotection is disputed [43,44]; however, it is perceived that such proteins may suppress pollen development by influencing cell cycle progression and phytohormone polarity [20,45]. Moreover, multiple orthologs to the Nod 19 protein (Nod19) in *Arabidopsis thaliana* L. were upregulated in stressed microspores. Nod19 proteins have not been reported in other androgenesis systems, although they are known to mitigate stress-generated oxidative damage in common bean (*Phaseolus vulgaris* L.; [46]). As such, their upregulation in this study was likely a consequence of low-temperature shock.

Cold stress also prompted an increased expression of *Glyma.11g250200*, which encodes a cytochrome P450 CYP78A5 monooxygenase. Orthologs to this gene are known gametophytic embryogenesis biomarkers in *Brassica napus* L. [20] and *Brassica campestris* L. [47]. Expression studies in *Arabidopsis thaliana* L. suggested that CYP78A5 is regulated downstream of other androgenesis-related sequences, including the Class I knotted-like homeodomain transcriptional regulator SHOOTMERISTEMLESS [20,48]. It is hypothesized that *Glyma.11g250200* is involved in stress response, signal transduction, and meristem initiation/identity, altogether encouraging the microspore transition toward embryogenesis [22,47,49].

### 2.3. Microspore Totipotency Was Initiated by Suppression of the Gametophytic Program

**Figure 2:**
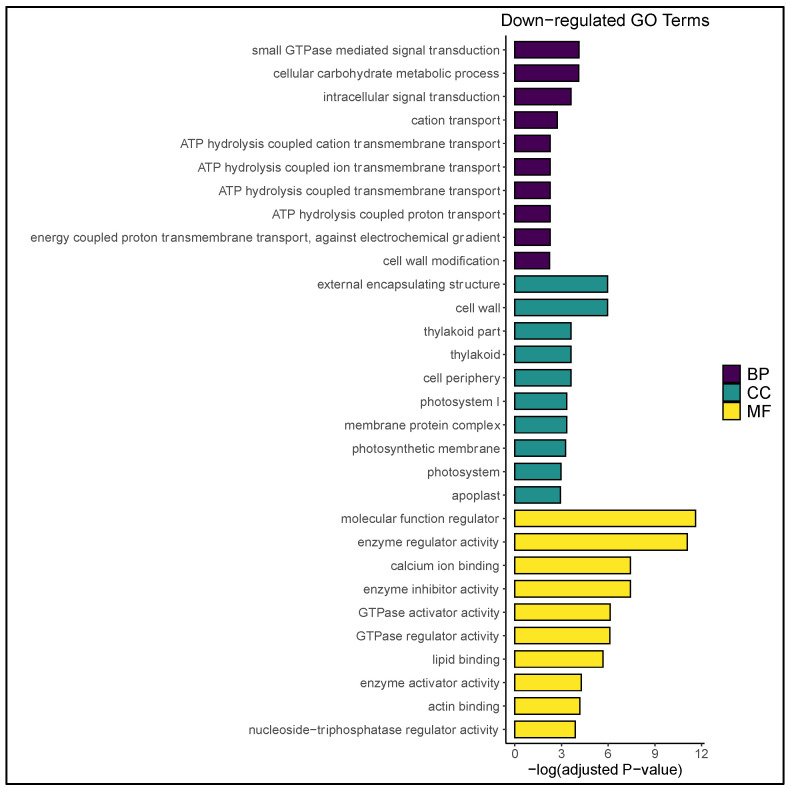
Downregulated gene ontology (GO) terms in the temperature-stressed microspore population with respect to the untreated control. Statistically significant biological processes (BP), cellular components (CC), and molecular functions (MF) are ranked in descending order by adjusted *p*-value (−log[adjusted *p*-value]). A comprehensive list of enriched GO terms is available in Supplementary File 2.

**Figure 3:**
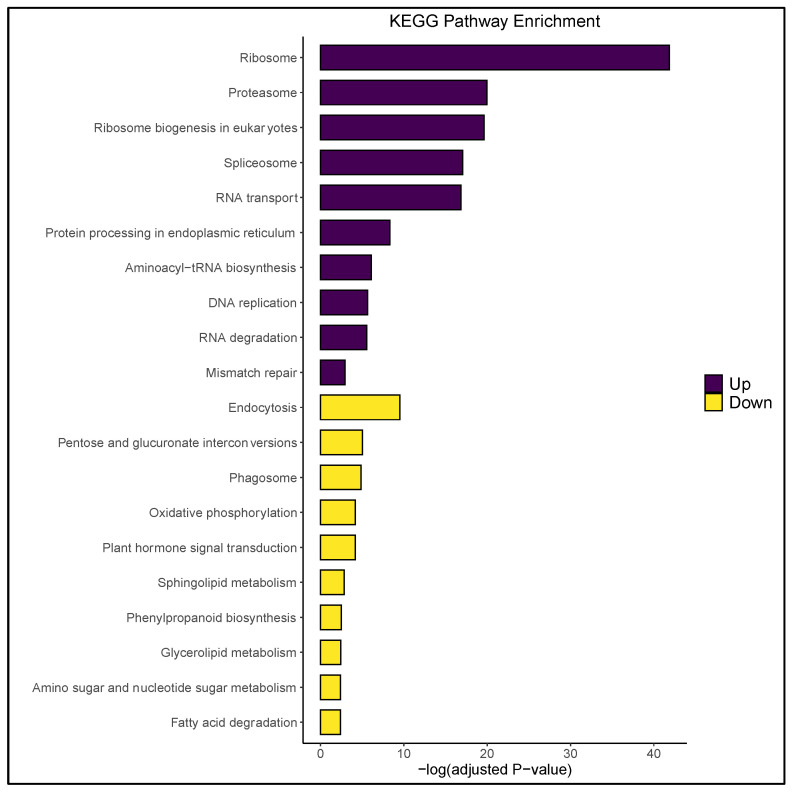
Kyoto Encyclopedia of Genes and Genomes (KEGG) pathway enrichment of differentially expressed genes (DEGs). Up- (purple) and downregulated (yellow) cellular pathways in temperature-stressed microspores with respect to the control population. A comprehensive list of enriched KEGG pathways is available in Supplementary File 3.

The expression profile of temperature-stressed microspores was characterized by a vast decrease in microgametogenesis-relevant transcripts. For example, prior work had demonstrated that pollen maturation requires increased expression of energy molecule-encoding genes to drive starch biosynthesis and pollen tube germination [50,51]. In this study, four gene ontology (GO)-enriched terms corresponding to adenosine triphosphate (ATP) hydrolysis-based transport were downregulated (Figure 2), as was the generation of electrochemical gradients that provide energy for the synthesis of ATP and guanosine-5′-triphosphate. These data suggested a significant reduction in downstream endergonic reactions as a consequence of stress, as well as altered purinergic signal transduction. In concurrence, KEGG (Kyoto Encyclopedia of Genes and Genomes) enrichment revealed oxidative phosphorylation as one of the most suppressed pathways in stressed microspores (Figure 3). These data are similar to the observations of Maraschin et al. [37] and Seifert et al. [1] in which an overall decrease in energy transport was associated with a developmental switch within the microspore.

It is well-established that microspore totipotency requires decreased biosynthesis of starch and lipid bodies and that excessive starch accumulation inhibits androgenic induction [3,9,52]. Such changes were reflected here by a decrease in overall carbohydrate metabolism, as well as the downregulation of saccharide and lipid metabolic processes (Figure 2 and Figure 3). This is in concert with GO enrichment terminology reported by Seifert et al. [1], as well as the aforementioned decrease in ATP hydrolysis-based transport, the latter of which may obstruct the reaction coupling potential required for starch/glycogen biosynthesis. Additionally, vacuolar-type H + -ATPase activity was suppressed in the stressed microspore population (Supplementary File 2), indicating decreased transport of osmotically active substances and inhibition of endomembrane acidification. This bears significance in the present context due to the reliance of pollen on vacuole-localized sucrose for starch anabolism (Figure 3; Supplementary File 2; [51]). These transcriptional changes likely precede the fragmented vacuole phenotype reported across numerous microspore embryogenesis platforms (Figure 1d; [7]).

**Figure 4:**
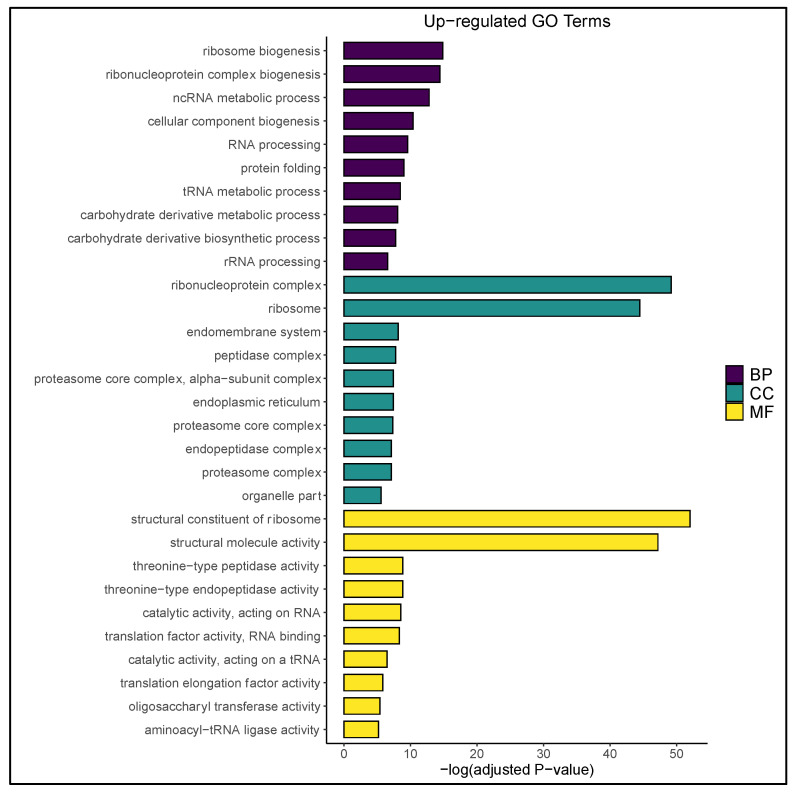
Upregulated GO terms in the temperature-stressed microspore population with respect to the untreated control. Statistically significant biological processes (BP), cellular components (CC), and molecular functions (MF) are ranked in descending order by adjusted *p*-value (−log[adjusted *p*-value]). A comprehensive list of enriched GO terms is available in Supplementary File 2.

Temperature stress enhanced the metabolism and biosynthesis of carbohydrate derivatives (Figure 4), including those produced by nonhydroxyl sugar modification. The “carbohydrate derivative metabolic process” GO category contained *Glyma.13g346700*, which is homologous to endochitinase protein3-3 (EP3-3)-encoding genes in *Arabidopsis thaliana* L. and carrot (*Daucus carota* L.). Moreover, the carrot EP3-3 chitinase has been demonstrated to influence cell fate determination during somatic embryogenesis via secretion from nonembryogenic cells and through the modification of arabinogalactan proteins. In both instances, an extracellular matrix was formed in the medium that improved the embryogenic response of the culture [9,53,54]. Chitinase- and arabinogalactan protein-rich matrices also proved crucial for embryo maturation in androgenic cultures of *Zea mays* L. [55]. Hence, increased expression of carbohydrate derivative-encoding genes supported the observation of matrix formation in soybean (Figure 1j,k) and suggested that the extruded substance contained arabinogalactan proteins and chitinases.

Further evidence for microspore dedifferentiation was demonstrated by an increase in proteolytic activity following stress treatment, a phenomenon previously shown to selectively degrade gametophytic proteins in *Hordeum vulgare* L. [9]. In soybean, this entailed the upregulation of multiple genes encoding members of the 20S proteosome complex (i.e., threonine-type endopeptidases), cytosolic aminopeptidases, ubiquitin carboxyl-terminal hydrolases, and other enzymes that reside in various peptidase complexes (Figure 3 and Figure 4, Supplementary File 1). Likewise, stressed microspores showed GO enrichment for ubiquitin-dependent protein catabolism (Supplementary File 2). These findings are in accordance with two other androgenesis-relevant RNA-Seq studies [1,22], as well as the gene chip work of Maraschin et al. [37]. Interestingly, endocytic and autophagic pathways were suppressed in the stressed microspore population (Figure 3). This contradicted the work of Bárány et al. [56] who used a similar stress regimen to induce autophagy in *Hordeum vulgare* L. microspores. It is possible that the stepwise application of temperature stress employed in the current study preserved microspore viability within the anther, mitigating the need for excessive homeostatic activity succeeding mechanical isolation. This hypothesis draws parallels to the callogenesis work of Garda et al. [25] and, if consistent with autophagy-inhibition studies in *Brassica napus* L. [57], illustrates that restricted expression of autophagic/endocytic pathways is essential to balance toxin accumulation/cell death in a manner conducive for microspore embryogenesis.

### 2.4. Stressed Microspores Expressed Transcripts that Evidenced the Onset of Sporophytic Growth

While the majority of stress-related gene expression pertained to microspore dedifferentiation, several DEG profiles implied the onset of an embryogenic developmental program. For example, GO enrichment showed decreased expression in pectinesterase activity following temperature stress (Supplementary File 2), characteristically suggesting increased esterification of galacturonic acid residues with methanol/acetic acid. An accumulation of esterified pectins within the exine/cell wall has been reported as a reliable biomarker for microspore embryogenesis in both *Capsicum annuum* L. [58] and cork oak (*Quercus suber* L. [59]). Furthermore, multiple genes regulating pectin methylesterase activity were differentially expressed, which bore a comparison to expression profiles in sporophytic microspores of *Brassica napus* L. [20] and *Brassica campestris* L. [47] (Supplementary File 1). Additional androgenesis-relevant cell wall modification was supported in this study by the upregulation of genes encoding exopolygalacturonases (i.e., *Glyma.10g016900*; [20]), arabinogalactan proteins (i.e., *Glyma.08g281600*; [60]), and pectate lyases (i.e., *Glyma.11g063300*; [20]) and an overall decrease in callose and cellulose biosynthesis (i.e., *Glyma.08g156800* and *Glyma.02g286100*, respectively) (Supplementary File 1). These data reinforced the work of Tang and Sun [10] in which a heightened frequency of exine dehiscence was observed when microspores were exposed to a 2–3 d cold pretreatment.

In *Triticum aestivum* L., it was demonstrated that establishment of the embryogenic program involved GO enrichment for terms related to transcription and translation, signifying a reset of the cell cycle [1]. Here, similar molecular functions were upregulated, including the “structural constituent of ribosome,” “translation factor activity, RNA binding,” and “translation elongation factor activity” (Figure 4), as well as genes controlling cell proliferation (i.e., *Glyma.13g096900*, which encodes the AP2 transcriptional regulator *AINTEGUMENTA-like 5*) (Supplementary File 1). Additionally, “Ribosome,” “RNA polymerase,” and “DNA replication” KEGG pathways were significantly increased in the stressed microspore population (Figure 3; Supplementary File 3). These results contradicted findings in microspore embryogenesis platforms of *Brassica napus* L. [20] and *Hordeum vulgare* L. [9,22] yet agreed with pollen stress response studies in *Arabidopsis thaliana* L. [61,62] and the soybean somatic embryogenesis work of Thibaud-Nissen et al. [63]. The trend in gene expression observed here suggested a degree of species/genotype specificity regarding temporal, stress-induced cell division/proliferation in microspores and pollen. In a like manner, genes encoding minichromosome maintenance proteins (MCM) were upregulated in the present study (Supplementary File 1). MCMs are components of DNA helicase that directly influence DNA replication and cell cycle progression [64]. Ni et al. [65] demonstrated the pertinence of MCM expression during early zygotic embryogenesis in *Arabidopsis thaliana* L., which was later postulated to be a key regulator of microspore embryogenesis induction in *Hordeum vulgare* L. [22]. The expression pattern of MCM-encoding genes in the latter study (down- then upregulated) suggests that MCMs represent the true expression of a new developmental program (totipotency/embryogenesis) rather than a stress-related response, and that temperature stress likely induces de-/redifferentiation within the soybean microspore.

## 3. Materials and Methods

### 3.1. Plant Materials

Seeds for soybean cultivar IAS-5 were obtained from the USDA Germplasm Resources Information Network and germinated in seed testing paper for 3 d in the dark. Eighty seedlings were transplanted into a Miracle Gro Moisture Control potting medium and grown in a Conviron growth chamber (Winnipeg, MB, Canada) at 28 °C continuous, 16 h d^−1^ light at 230 to 365 μM m^−2^ s^−1^, 90% relative humidity. Prior to anthesis, half of the plants were subjected to low-temperature stress (10 °C day/8 °C night for 3 d in a Conviron chamber followed by 4 °C for 14–16 h in a refrigerator [31]) and the other half maintained under normal growth conditions. Floral bud selection and dissection were performed the following day.

### 3.2. Floral Bud Selection and Microspore Isolation

Studies have demonstrated that 4 mm IAS-5 floral buds grown in a controlled environment contain >90% late unicellular-bicellular microspores; thus, this developmental stage was selected for all experiments [25,31]. In so doing, buds were measured from the base of the calyx to the tip of the uppermost sepal. For each treatment condition (stress and no stress), 120 floral buds were selected. Prior to microspore isolation, each treatment was subdivided into 4 technical replicates, each comprised of 30 buds.

Buds were sterilized in a 20% bleach solution (active ingredient 6% NaClO) for 7.5 min and rinsed thrice with sterile water. Androecia were then isolated with the use of a Zeiss™ Stemi 2000-C Stereo Microscope (Jena, Germany) and anthers suspended in a 0.4 M mannitol solution. Microspores were released by squashing anthers with a glass rod. Contaminating tissues were removed from the microspore slurry with 60 and 41 μm vacuum-driven filtration systems. Additional filtration was performed with a 40 μm cell strainer. Following purification, 15 μL samples were collected to estimate microspore density across replicates. Replicates were then centrifuged for 6 min at 2000 RPM, supernatant was discarded, and pelleted microspores were frozen in liquid nitrogen and stored at −80 °C.

To investigate the progression of in vitro embryogenesis, additional populations of temperature-stressed microspores were generated and suspended in a liquid BNN induction medium [25] supplemented with 10 mg L^−1^ 2,4-dichlorophenoxyacetic acid, 0.1 mg L^−1^ N^6^-benzyladenine, 2% sucrose, and 2% sorbitol. On day 15, microspores were subcultured into a liquid BABI medium [66] plus 0.35 mg L^−1^ *N*^6^-benzyladenine and 6.0 μg L^−1^ picloram. Incubation conditions entailed a 3 d acclimatization period at 11 °C, no light, in an Innova shaker (New Brunswick Scientific; Edison, NJ, USA). Cultures were then moved to a Conviron chamber set at 18 °C with minimal light intensity (99 to 111 μM m^−2^ s^−1^; 16 h d^−1^) for days 4–7. From day 8 onward, microspore cultures were kept in a chamber at 25 °C with maintained light.

### 3.3. Estimation of Microspore Density

Microspore density was estimated using an Invitrogen™ Countess™ automated cell counter (Carlsbad, CA, USA). The 15 μL samples were first mixed with 0.4% Trypan Blue (ThermoFisher Cat # T10282; Waltham, MA, USA). Homogenous mixtures were loaded onto cell counting chamber slides (Invitrogen™ Cat # C10228) and analyzed as described previously [25]. All readings were performed in triplicate.

### 3.4. Sample Preparation and Microscopy

Cultured microspores were observed periodically using light and fluorescent microscopy. In both instances, the cellular fixation of microspores was performed through culture centrifugation followed by resuspension and incubation in 4% paraformaldehyde in phosphate-buffered saline (PBS), pH 7.4, overnight at 4 °C. For light microscopy, fixed microspores were rinsed thrice in PBS and immediately mounted on 2% gelatin-coated glass slides. Image capture and processing were performed with a BioTek Lionheart FX Automated Microscope (Winooski, VT, USA) equipped with Gen5 Image + software (v3.08). Embryogenic structures ≥1 mm and unfixed, whole-culture suspensions were analyzed with a Zeiss™ Stemi 2000-C Stereo Microscope (Oberkochen, Germany) paired with ZEN Imaging software (v2.3 [blue edition]).

Developmental stage and mitotic division symmetry were visualized by incubating fixed microspores in citrate-phosphate buffer (bioWORLD; Cat # 40120281-1; Dublin, OH, USA) containing 5 μg mL^−1^ 4′6-diamidino-2-phenylindole (DAPI) (ThermoFisher Cat # D1306) and 1% Triton X-100 (Sigma-Aldrich Cas # 9002-93-1, St. Louis, MO, USA) for 10 min. Samples were rinsed three times with PBS and mounted on 2% gelatin-coated glass slides. The BioTek Lionheart FX Automated Microscope was used for all fluorescent microscopy with laser excitation/emission wavelengths of 365/447 nm, respectively. Images were processed as described for light microscopy.

### 3.5. RNA Isolation, Quantification, and Qualification

Total RNA was isolated from microspores using the Zymo Research Quick-RNA Microprep Kit (Cat # R1050; Irvine, CA, USA) as per the manufacturer’s protocol with modifications (RNA elution with 25 μL DNase/RNase free water). Total RNA concentration was measured with the Thermo Scientific™ NanoDrop™ OneC Spectrophotometer (Waltham, MA, USA). RNA purity was estimated from the OD 260/280 and the 260/230 ratios. Additionally, RNA integrity was evaluated using the RNA Nano 6000 Assay Kit of the Bioanalyzer 2100 system (Agilent Technologies, Santa Clara, CA, USA). Samples with an RNA integrity number (RIN) ≥ 7 were considered for library construction and sequencing. Subsequent library preparation, sequencing, and data analyses were performed at Novogene Corporation Inc. (Sacramento, CA, USA).

### 3.6. cDNA Library Preparation

Approximately 100 ng RNA per replicate was used as input material for library preparation. Sequencing libraries were generated using NEBNext^®^ Ultra™ RNA Library Prep Kit for Illumina^®^ (Cat # E7530S; New England Biolabs, Ipswich, MA, USA) following the manufacturer’s recommendations. Index codes were added to attribute sequences to each replicate. Briefly, mRNA was purified from total RNA using poly-T oligo-attached magnetic beads. Fragmentation was carried out using divalent cations under elevated temperature in NEBNext First Strand Synthesis Reaction Buffer (5X). First-strand cDNA was synthesized using a random hexamer primer and M-MuLV Reverse Transcriptase (RNase H-). Second-strand cDNA synthesis was subsequently performed using DNA Polymerase I and RNase H. Remaining overhangs were converted into blunt ends via exonuclease/polymerase activities. After adenylation of the 3′ ends of DNA fragments, NEBNext Adaptors with a hairpin loop structure were ligated to prepare for hybridization. In order to select cDNA fragments of preferentially 150–200 base pairs in length, the library fragments were purified with the AMPure XP system (Cat # A63881; Beckman Coulter, Brea, CA, USA). Next, 3 µL of NEB USER^®^ Enzyme (Cat # M5505S, New England Biolabs, Ipswich, MA, USA) was used with size-selected, adaptor-ligated cDNA at 37 °C for 15 min followed by 5 min at 95 °C. Polymerase chain reaction (PCR) was then performed with Phusion High-Fidelity DNA polymerase, universal PCR primers, and index primers. Lastly, PCR products were purified (AMPure XP system, Beckman Coulter, Indianapolis, IN, USA) and library quality was assessed on the Agilent Bioanalyzer 2100 system.

### 3.7. Cluster Generation and Sequencing

Clustering of the index-coded replicates was performed on a cBot Cluster Generation System using PE Cluster Kit cBot-HS (Cat # PE-401-3001; Illumina, San Diego, CA, USA) according to the manufacturer’s instructions. After cluster generation, the library preparations were sequenced on an Illumina HiSeq2500 platform and paired-end reads were generated.

### 3.8. Data Processing, Analyses, and Visualization

Raw reads of FASTQ format were analyzed and preprocessed using fastp software (v0.20.1) [67]. During this step, poly-N sequences and low-quality reads (Q value ≤ 20) were removed from raw data. Resultant clean reads were mapped to the whole-genome sequence of the Williams82 reference genome (v2.0.40; ftp://ftp.ensemblgenomes.org/pub/release-40/plants/gtf/glycine_max/) using HISAT2 software (v2.2.0) [68]. StringTie (v2.1.2) [69] was used to assemble the set of transcript isoforms of each BAM file obtained in the mapping step. FeatureCounts (v2.0.0) [70] was then used to count the read numbers mapped of each gene, including known and novel genes. Reads Per Kilobase of exon model per Million mapped reads (RPKM) was calculated based on the length of the gene and reads count mapped to this gene.

Differential expression analysis between cold shock and control samples was performed using the DESeq2 package (v1.28.1) [71] within R statistical software [72] with row count data as input. Resulting *p*-values were adjusted using the Benjamini–Hochberg method for controlling false discovery rate. Following normalization, genes with an adjusted *p*-value < 0.05 were assigned as differentially expressed. Hierarchical clustering of samples was performed based on Pearson’s correlation coefficients.

Differentially expressed genes (DEGs) were subjected to functional enrichment analyses. Gene ontology (GO) enrichment was performed with the ClusterProfiler R package (v3.16.1) [73] in which gene length bias was corrected. GO terms with a corrected *p*-value < 0.05 were considered significantly enriched by DEGs. Additionally, DEGs were annotated to cellular pathways using the Kyoto Encyclopedia of Genes and Genomes (KEGG) [74]. ClusterProfiler was used to test the statistical enrichment of genes in KEGG pathways. DEGs were then compared to orthologous sequences in *Arabidopsis thaliana* L. (https://www.arabidopsis.org/). All functional enrichment analyses were visualized using the ggplot2 package (v3.3.2) [75] within R.

### 3.9. Data Availability

Raw sequence data generated in this study have been submitted to the NCBI’s Sequence Read Archive (Submission #SUB8220792).

## 4. Conclusions

This study demonstrates the first high-throughput transcriptome analysis in a nonmodel androgenesis system. The key transcriptional shift in stressed soybean microspores entailed suppression of the microgametophytic program, characteristically implying the onset of microspore reprogramming. Concurrently, molecular programs corresponding to stress response and embryo induction were upregulated. Altogether, these expression profiles confirmed that pretreatment temperature stress was sufficient to divert soybean microspores from gametophytic to sporophytic development, similar to model organisms, thus providing a functional framework for nonzygotic embryo ontogeny. Future research should investigate the transcriptional landscape of dividing embryogenic masses with respect to the microspore, as well as distinguish stress-related genes from those that regulate embryogenesis.

## Supplementary Materials

The following are available online at https://www.mdpi.com/2223-7747/9/11/1510/s1, File 1: DEG list; File 2: Up- and downregulated significantly enriched GO terms in the stressed microspore population; File 3: Up- and downregulated significantly enriched KEGG pathways in the stressed microspore population.

## Abbreviations:

ATP	adenosine triphosphate
DAPI	4′6-diamidino-2-phenylindole
DEG	differentially expressed gene
EP3-3	endochitinase protein3-3
GO	gene ontology
HSP	heat shock protein
KEGG	Kyoto Encyclopedia of Genes and Genomes
MCM	minichromosome maintenance protein
PBS	Phosphate-buffered saline
PCR	polymerase chain reaction
RIN	RNA integrity number
RNA	ribonucleic acid
RNA-Seq	RNA sequencing
RPKM	reads per kilobase of exon model per million mapped reads
Srna	small RNA

## References

[1] Seifert F., Bössow S., Kumlehn J., Gnad H., Scholten S. (2016). Analysis of wheat microspore embryogenesis induction by transcriptome and small RNA sequencing using the highly responsive cultivar “Svilena”. BMC Plant Biol..

[2] Seguí-Simarro J.M. (2010). Androgenesis revisited. Bot. Rev..

[3] Touraev A., Vicente O., Heberle-Bors E. (1997). Initiation of microspore embryogenesis by stress. Trends Plant Sci..

[4] Germanà M.A. (2011). Gametic embryogenesis and haploid technology as valuable support to plant breeding. Plant Cell Rep..

[5] Maluszynski M., Kasha K.J., Szarejko I., Maluszynski M., Kasha K.J., Forster B.P., Szarejko I. (2003). Published Doubled Haploid Protocols in Plant Species. Doubled Haploid Production in Crop Plants: A Manual.

[6] Twell D., Park S.K., Lalanne E. (1998). Asymmetric division and cell-fate determination in developing pollen. Trends Plant Sci..

[7] Shariatpanahi M.E., Bal U., Heberle-Bors E., Touraev A. (2006). Stresses applied for the reprogramming of plant microspores towards in vitro embryogenesis. Physiol. Plant.

[8] Touraev A., Indrianto A., Wratschko I., Vicente O., Heberle-Bors E. (1996). Efficient microspore embryogenesis in wheat (*Triticum aestivum* L.) induced by starvation at high temperature. Sex. Plant Rep..

[9] Maraschin S.D.F., De Priester W., Spaink H.P., Wang M. (2005). Androgenic switch: An example of plant embryogenesis from the male gametophyte perspective. J. Exp. Bot..

[10] Tang X.C., Sun M.X. (2007). Exine-dehisced microspores: A novel model system for studying embryogenesis. Int. J. Plant Dev. Biol..

[11] Song H., Lou Q.F., Luo X.D., Wolukau J.N., Diao W.P., Qian C.T., Chen J.F. (2007). Regeneration of doubled haploid plants by androgenesis of cucumber (*Cucumis sativus* L.). Plant Cell Tissue Organ. Cult..

[12] Testillano P.S. (2019). Microspore embryogenesis: Targeting the determinant factors of stress-induced cell reprogramming for crop improvement. J. Exp. Bot..

[13] Pérez-Pérez Y.O., El-Tantawy A.A., González M.S., Risueno M.C., Testillano P.S. (2019). Stress-induced microspore embryogenesis requires endogenous auxin synthesis and polar transport in barley. Front. Plant Sci..

[14] Rivas-Sendra A., Calabiug-Serna A., Seguí-Simarro J.M. (2017). Dynamics of calcium during in vitro microspore embryogenesis and in vivo microspore development in *Brassica napus* and *Solanum melongena*. Front. Plant Sci..

[15] Pauls K.P., Chan J., Woronuk G., Schulze D., Brazolot J. (2006). When microspores decide to become embryos—Cellular and molecular changes. Can. J. Bot..

[16] Belanger S., Baldrich P., Lemay M.A., Marchand S., Esteves P., Meyers B.C., Belzile F.C. (2020). The commitment of barley microspores into embryogenesis involves miRNA-directed regulation of members of the SPL, GRF and HD-ZIPIII transcription factor families. bioRxiv.

[17] Solís M.T., El-Tantawy A.A., Cano V., Risueño M.C., Testillano P.S. (2015). 5-azacytidine promotes microspore embryogenesis initiation by decreasing global DNA methylation, but prevents subsequent embryo development in rapeseed and barley. Front. Plant Sci..

[18] Berenguer E., Bárány I., Solís M.T., Pérez-Pérez Y., Risueño M.C., Testillano P.S. (2017). Inhibition of histone H3K9 methylation by BIX-01294 promotes stress-induced microspore totipotency and enhances embryogenesis initiation. Front. Plant Sci..

[19] Li H., Soriano M., Cordewener J., Muiño J.M., Riksen T., Fukuoka H., Angenent G.C., Boutilier K. (2014). The histone deacetylase inhibitor trichostatin a promotes totipotency in the male gametophyte. Plant Cell.

[20] Malik M.R., Wang F., Dirpaul J.M., Zhou N., Polowick P.L., Ferrie A.M., Krochko J.E. (2007). Transcript profiling and identification of molecular markers for early microspore embryogenesis in *Brassica napus*. Plant Phys..

[21] Soriano M., Li H., Boutilier K. (2013). Microspore embryogenesis: Establishment of embryo identity and pattern in culture. Plant Rep..

[22] Bélanger S., Marchand S., Jacques P.É., Meyers B., Belzile F. (2018). Differential expression profiling of microspores during the early stages of isolated microspore culture using the responsive barley cultivar gobernadora. G3 Genes Genomes Genet..

[23] Corral-Martínez P., Siemons C., Horstman A., Angenent G.C., de Ruijter N., Boutilier K. (2020). Live Imaging of embryogenic structures in *Brassica napus* microspore embryo cultures highlights the developmental plasticity of induced totipotent cells. Plant Rep..

[24] Lulsdorf M.M., Croser J.S., Ochatt S. (2011). 11 Androgenesis and Doubled-Haploid Production in Food Legumes. Biol. Breed. Food Legumes.

[25] Garda M., Hale B., Rao N., Lowe M., Bright M., Goodling S., Phillips G.C. (2020). Soybean androgenesis I: Identification of pyramidal stressors in anther cultures that sustain cell divisions and putative embryo formation from isolated microspore cultures. Vitro Cell Dev. Biol. Plant.

[26] Nitsch J.P., Nitsch C. (1969). Haploid plants from pollen grains. Science.

[27] Gamborg O.L., Miller R., Ojima K. (1968). Nutrient requirements of suspension cultures of soybean root cells. Exp. Cell Res..

[28] Lichter R. (1982). Induction of haploid plants from isolated pollen of *Brassica napus*. Z Pflanzenphysiol..

[29] Ranch J.P. (1986). Plant regeneration from tissue cultures of soybean by somatic embryogenesis. Cell Cult. Somat. Cell Genet. Plants.

[30] Phillips G.C., Collins G.B. (1979). In vitro tissue culture of selected legumes and plant regeneration from callus cultures of red clover. Crop Sci..

[31] Hale B., Phipps C., Rao N., Kelley C., Phillips G.C. (2020). Soybean androgenesis II: Characterization of early embryogenesis from isolated microspore cultures. Vitro Cell Dev. Biol. Plant.

[32] Ferrie A.M.R., Caswell K.L. (2011). Isolated microspore culture techniques and recent progress for haploid and doubled haploid plant production. Plant Cell Tissue Organ. Cult..

[33] Rodrigues L.R., Oliveira J.M.S., Mariath J.E., Bodanese-Zanettini M.H. (2005). Histology of embryogenic responses in soybean anther culture. Plant Cell Tissue Organ. Cult..

[34] Moraes A.P.D., Bonadese-Zanettini M.H., Callegari-Jacques S.M., Kaltchuk-Santos E. (2004). Effect of temperature shock on soybean microspore embryogenesis. Braz. Arch. Biol. Technol..

[35] Kaltchuk-Santos E., Mariath J.E., Mundstock E., Hu C.Y., Bodanese-Zanettini M.H. (1997). Cytological analysis of early microspore divisions and embryo formation in cultured soybean anthers. Plant Cell Tissue Organ. Cult..

[36] Kaltchuk-Santos E., Zanettini M.H.B., Mundstock E. (1993). Pollen dimorphism in soybean. Protoplasma.

[37] Maraschin S.D.F., Caspers M., Potokina E., Wülfert F., Graner A., Spaink H.P., Wang M. (2006). cDNA array analysis of stress-induced gene expression in barley androgenesis. Phys. Plant.

[38] Joosen R., Cordewener J., Supena E.D.J., Vorst O., Lammers M., Maliepaard C., Zeilmaker T., Miki B., America T., Custers J. (2007). Combined transcriptome and proteome analysis identifies pathways and markers associated with the establishment of rapeseed microspore-derived embryo development. Plant Phys..

[39] Muñoz-Amatriaín M., Svensson J.T., Castillo A.M., Close T.J., Vallés M.P. (2009). Microspore embryogenesis: Assignment of genes to embryo formation and green vs. albino plant production. Funct. Int. Genom..

[40] Seguí-Simarro J.M., Testillano P.S., Risueno M.C. (2003). Hsp70 and Hsp90 change their expression and subcellular localization after microspore embryogenesis induction in *Brassica napus* L.. J. Struct. Biol..

[41] Bárány I., Testillano P.S., Mitykó J., Risueno M.C. (2001). The switch of the microspore program in Capsicum involves HSP70 expression and leads to the production of haploid plants. Int. J. Dev. Biol..

[42] Zarsky V., Garrido D., Eller N., Tupy J., Vicente O., Schöffl F., Heberle-Bors E. (1995). The expression of a small heat shock gene is activated during induction of tobacco pollen embryogenesis by starvation. Plant Cell Environ..

[43] Telmer C.A., Newcomb W., Simmonds D.H. (1995). Cellular changes during heat shock induction and embryo development of cultured microspores of *Brassica napus* cv. Topas. Protoplasma.

[44] Zhao J., Newcomb W., Simmonds D. (2003). Heat-shock proteins 70 kDa and 19 kDa are not required for induction of embryogenesis of *Brassica napus* L. cv. Topas microspores. Plant Cell Phys..

[45] Dubas E., Custers J., Kieft H., Wędzony M., van Lammeren A.A. (2014). Characterization of polarity development through 2-and 3-D imaging during the initial phase of microspore embryogenesis in *Brassica napus* L.. Protoplasma.

[46] Naya L., Paul S., Valdés-López O., Mendoza-Soto A.B., Nova-Franco B., Sosa-Valencia G., Reyes J.L., Hernández G. (2014). Regulation of copper homeostasis and biotic interactions by microRNA 398b in common bean. PLoS ONE.

[47] Zhang Y., Gao S.Y., Liu H.H., Zhang X.L., Zeng A.S., Wang J.J., Hou X.L., Li Y. (2020). cDNA-AFLP analysis of differentially expressed genes during microspore embryogenesis in non-heading Chinese cabbage. Vitro Cell Dev. Biol. Plant.

[48] Zondlo S.C., Irish V.F. (1999). CYP78A5 encodes a cytochrome P450 that marks the shoot apical meristem boundary in Arabidopsis. Plant J..

[49] Ehlting J., Sauveplane V., Olry A., Ginglinger J.F., Provart N.J., Werck-Reichhart D. (2008). An extensive (co-) expression analysis tool for the cytochrome P450 superfamily in Arabidopsis thaliana. BMC Plant Biol..

[50] McCormick S. (1993). Male gametophyte development. Plant Cell.

[51] Datta R., Chourey P.S., Pring D.R., Tang H.V. (2001). Gene-expression analysis of sucrose-starch metabolism during pollen maturation in cytoplasmic male-sterile and fertile lines in sorghum. Sex. Plant Rep..

[52] Hosp J., Maraschin S.F.D., Touraev A., Boutilier K. (2007). Functional genomics of microspore embryogenesis. Euphytica.

[53] van Hengel A.J., Guzzo F., van Kammen A., de Vries S.C. (1993). Expression pattern of the carrot EP3 endochitinase genes in suspension cultures and in developing seeds. Plant Phys..

[54] van Hengel A.J., Tadesse Z., Immerzeel P., Schols H., Van Kammen A.B., de Vries S.C. (2001). N-acetylglucosamine and glucosamine-containing arabinogalactan proteins control somatic embryogenesis. Plant Phys..

[55] Borderies G., Le Béchec M., Rossignol M., Lafitte C., Le Deunff E., Beckert M., Dumas C., Matthys-Rochon E. (2004). Characterization of proteins secreted during maize microspore culture: Arabinogalactan proteins (AGPs) stimulate embryo development. Eur. J. Cell Biol..

[56] Bárány I., Berenguer E., Solís M.T., Pérez-Pérez Y., Santamaría M.E., Crespo J.L., Risueño M.C., Díaz I., Testillano P.S. (2018). Autophagy is activated and involved in cell death with participation of cathepsins during stress-induced microspore embryogenesis in barley. J. Exp. Bot..

[57] Berenguer E., Minina E.A., Carneros E., Bárány I., Bozhkov P.V., Testillano P.S. (2020). Suppression of Metacaspase-and Autophagy-Dependent Cell Death Improves Stress-Induced Microspore Embryogenesis in Brassica Napus. http://hdl.handle.net/10261/221508.

[58] Bárány I., Fadón B., Risueño M.C., Testillano P.S. (2010). Cell wall components and pectin esterification levels as markers of proliferation and differentiation events during pollen development and pollen embryogenesis in *Capsicum annuum* L.. J. Exp. Bot..

[59] Rodríguez-Sanz H., Manzanera J.A., Solís M.T., Gómez-Garay A., Pintos B., Risueño M.C., Testillano P.S. (2014). Early markers are present in both embryogenesis pathways from microspores and immature zygotic embryos in cork oak, *Quercus suber* L.. BMC Plant Biol..

[60] El-Tantawy A.A., Solís M.T., Da Costa M.L., Coimbra S., Risueño M.C., Testillano P.S. (2013). Arabinogalactan protein profiles and distribution patterns during microspore embryogenesis and pollen development in *Brassica napus*. Plant Rep..

[61] Lee J.Y., Lee D.H. (2003). Use of serial analysis of gene expression technology to reveal changes in gene expression in Arabidopsis pollen undergoing cold stress. Plant Phys..

[62] Honys D., Twell D. (2003). Comparative analysis of the Arabidopsis pollen transcriptome. Plant Phys..

[63] Thibaud-Nissen F., Shealy R.T., Khanna A., Vodkin L.O. (2003). Clustering of microarray data reveals transcript patterns associated with somatic embryogenesis in soybean. Plant Phys..

[64] Chong J.P., Hayashi M.K., Simon M.N., Xu R.M., Stillman B. (2000). A double-hexamer archaeal minichromosome maintenance protein is an ATP-dependent DNA helicase. Proc. Nat. Acad. Sci. USA.

[65] Ni D.A., Sozzani R., Blanchet S., Domenichini S., Reuzeau C., Cella R., Bergounioux C., Raynaud C. (2009). The Arabidopsis MCM2 gene is essential to embryo development and its over-expression alters root meristem function. New Phytol..

[66] Greenway M.B., Phillips I.C., Lloyd M.N., Hubstenberger J.F., Phillips G.C. (2012). A nutrient medium for diverse applications and tissue growth of plant species in vitro. Vitro Cell Dev. Biol. Plant.

[67] Chen S., Zhou Y., Chen Y., Gu J. (2018). fastp: An ultra-fast all-in-one FASTQ preprocessor. Bioinformatics.

[68] Kim D., Paggi J.M., Park C., Bennett C., Salzberg S.L. (2019). Graph-based genome alignment and genotyping with HISAT2 and HISAT-genotype. Nat. Biotechnol..

[69] Pertea M., Pertea G.M., Antonescu C.M., Chang T.C., Mendell J.T., Salzberg S.L. (2015). StringTie enables improved reconstruction of a transcriptome from RNA-seq reads. Nat. Biotechnol..

[70] Liao Y., Smyth G.K., Shi W. (2014). featureCounts: An efficient general purpose program for assigning sequence reads to genomic features. Bioinformatics.

[71] Love M.I., Huber W., Anders S. (2014). Moderated estimation of fold change and dispersion for RNA-seq data with DESeq2. Genome Biol..

[72] R Core Team (2017). R: A language and environment for statistical computing. R Found. Stat. Comp..

[73] Yu G. (2018). clusterProfiler: Universal enrichment tool for functional and comparative study. BioRxiv.

[74] Kanehisa M., Goto S. (2000). KEGG: Kyoto encyclopedia of genes and genomes. Nucleic Acids Res..

[75] Wickham H. (2016). Ggplot2: Elegant Graphics for Data Analysis.

