# Correction: Hale et al. Differential Expression Profiling Reveals Stress-Induced Cell Fate Divergence in Soybean Microspores. *Plants* 2020, *9*, 1510

**DOI:** 10.3390/plants13131881

**Published:** 2024-07-08

**Authors:** Brett Hale, Callie Phipps, Naina Rao, Asela Wijeratne, Gregory C. Phillips

**Affiliations:** 1 College of Science and Mathematics, Arkansas State University, Jonesboro, AR 72467-1080, USA; awijeratne@astate.edu; 2 Arkansas Biosciences Institute, Arkansas State University, Jonesboro, AR 72467-0639, USA; callie.phipps@smail.astate.edu (C.P.); naina.rao@outlook.com (N.R.); gphillips@astate.edu (G.C.P.); 3 College of Agriculture, Arkansas State University, Jonesboro, AR 72467-1080, USA; 4 Agricultural Experiment Station, University of Arkansas System Division of Agriculture, Jonesboro, AR 72467-2340, USA

In the original publication [1], there was a mistake in Figure 1 as published. Panels d and e were not soybean, but rather a second plant species that was being studied simultaneously. The corrected Figure 1 appears below. 

The authors state that the scientific conclusions are unaffected. This correction was approved by the Academic Editor. The original publication has also been updated.

## Figures and Tables

**Figure 1:**
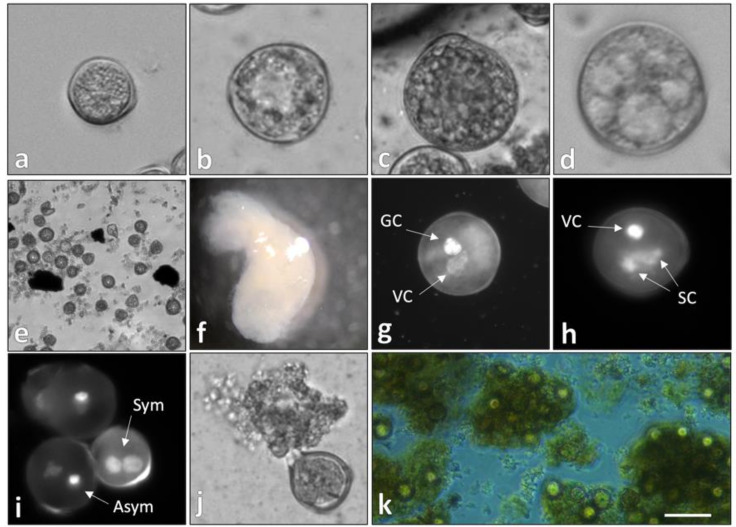
In vitro development of temperature-stressed soybean microspores. (**a**–**c**) Microgametogenesis, denoted by cell enlargement and the presence of starch granules; (**d**–**f**) microspore embryogenesis in soybean. (**d**) Embryogenic microspore with evident cytoskeletal fibers fragmenting the cytoplasm; (**e**) rapidly dividing pro-embryo; (**f**) microspore-derived embryo with established polarity; (**g**,**h**) pollen mitosis 1 (**g**) and 2 (**h**) observed in noninduced microspores; (**i**–**k**) cytological markers associated with an embryogenic culture. (**i**) Symmetrical division during pollen mitosis 1 observed via DAPI staining; (**j**,**k**) secretion of intrinsic molecules from nonisodiametric cells into the induction medium, forming a matrix. VC = vegetative cell; GC = generative cell; SC = sperm cell; Sym = symmetrical mitotic division; Asym = asymmetrical mitotic division. (**a**–**c**) bars = 15 μm; (**d**) 5 μm; (**e**) 100 μm; (**f**) 1 mm; (**g**,**h**) 10 μm; (**i**,**j**) 15 μm; (**k**) 100 μm.
